# Exploratory metabolomics study of the experimental opisthorchiasis in a laboratory animal model (golden hamster, *Mesocricetus auratus*)

**DOI:** 10.1371/journal.pntd.0006044

**Published:** 2017-10-31

**Authors:** Daria A. Kokova, Sarantos Kostidis, Judit Morello, Nataly Dementeva, Ekaterina A. Perina, Vladimir V. Ivanov, Ludmila M. Ogorodova, Aleksey E. Sazonov, Irina V. Saltykova, Oleg A. Mayboroda

**Affiliations:** 1 Department of Parasitology, Leiden University Medical Center, Leiden, The Netherlands; 2 Center for Proteomics and Metabolomics, Leiden University Medical Centre, Leiden, The Netherlands; 3 Translational Pharmacology, Chronic Diseases Research Center, NOVA Medical School, Lisbon, Portugal; 4 Department of Chemistry, Tomsk State University, Tomsk, Russian Federation; 5 Central Research Laboratory Siberian State Medical University, Tomsk, Tomsk, Russian Federation; 6 Department of Faculty Pediatrics, Siberian State Medical University, Tomsk, Tomsk, Russian Federation; Queen's University Belfast, UNITED KINGDOM

## Abstract

**Background:**

Opisthorchiasis is a parasitic infection caused by the liver flukes of the Opisthorchiidae family. Both experimental and epidemiological data strongly support a role of these parasites in the etiology of the hepatobiliary pathologies and an increased risk of intrahepatic cholangiocarcinoma. Understanding a functional link between the infection and hepatobiliary pathologies requires a detailed description a host-parasite interaction on different levels of biological regulation including the metabolic response on the infection. The last one, however, remains practically undocumented. Here we are describing a host response on Opisthorchiidae infection using a metabolomics approach and present the first exploratory metabolomics study of an experimental model of *O*. *felineus* infection.

**Methodology and Principal findings:**

We conducted a Nuclear Magnetic Resonance (NMR) based longitudinal metabolomics study involving a cohort of 30 animals with two degrees of infection and a control group. An exploratory analysis shows that the most noticeable trend (30% of total variance) in the data was related to the gender differences. Therefore further analysis was done of each gender group separately applying a multivariate extension of the ANOVA—ASCA (ANOVA simultaneous component analysis). We show that in the males the infection specific time trends are present in the main component (43.5% variance), while in the females it is presented only in the second component and covers 24% of the variance. We have selected and annotated 24 metabolites associated with the observed effects and provided a physiological interpretation of the findings.

**Conclusions:**

The first exploratory metabolomics study an experimental model of *O*. *felineus* infection is presented. Our data show that at early stage of infection a response of an organism unfolds in a gender specific manner. Also main physiological mechanisms affected appear rather nonspecific (a status of the metabolic stress) the data provides a set of the hypothesis for a search of the more specific metabolic markers of the Opisthorchiidae infection.

## Author summary

*Opisthorchiasis* is a parasitic infection caused by the liver flukes of the Opisthorchiidae family (*Trematoda*; *Platyhelminthes*). The liver fluke infections trigger development of the hepatobiliary pathologies such as chronic forms of cholecystitis, cholangitis, pancreatitis, and cholelithiasis. However, the most threatening effect of the Opisthorchiidae infection is an increased risk of intrahepatic cholangiocarcinoma. With this work we are getting an insight into a host response on Opisthorchiidae infection using a metabolomics approach. Metabolomics is a post-genomic discipline studying the metabolome. The dynamic character of the metabolome, its ability to change in response to the external stimuli makes it an optimal “readout” for exploratory studies aiming for the description of the systemic responses of an organism. Using this approach we demonstrate that that early response to the *O*. *felineus* infection unfolds in a gender-dependent manner. Moreover, with this first exploratory analysis of the metabolic response to *O*. *felineus* infection in an animal model we present a subset of the metabolites changing during the early phase of the infection and offer a possible physiological interpretation.

## Introduction

*Opisthorchiasis* is a parasitic infection caused by the liver flukes of the Opisthorchiidae family (*Trematoda*; *Platyhelminthes*). The family includes the three most important species for human health: *C*. *sinensis*, *O*. *viverrini*, *O*. *felineus*; together they are responsible for more than 45 million infections worldwide; 600–750 million people are currently at risk [1, 2]. *O*. *viverrini* and *C*. *sinensis* are endemic to the Far East regions and South East Asia remaining an important public health problem [3]. *O*. *felineus* infection is highly prevalent in Eastern Europe (Ukraine and the European part of Russia), Central Asia (northern Kazakhstan) and North Asia (Siberia) [2]. Both experimental and epidemiological data strongly support a role of liver fluke infections in the etiology of the hepatobiliary pathologies such as chronic forms of cholecystitis, cholangitis, pancreatitis, and cholelithiasis [2–5]. However, the most threatening effect of the Opisthorchiidae infection is an increased risk of intrahepatic cholangiocarcinoma [6].

Unlike other pathogens, *e*.*g*. viruses and microbes, parasitic helminthes do not proliferate within their mammalian host. Accordingly, the intensity of infection is one of the leading causes of the morbidity. For instance, *O*. *viverrini* associated symptoms occurred more frequently in those with high intensities of infection [7] and positive association between hepatobiliary pathology and *O*. *viverrini* infection intensity is well documented [4]. Moreover, the biliary tract abnormalities more often detected by ultrasonography in the patients with heavy *O*. *viverrini* infections than light or moderate ones [8]. However, despite clear phenomenological and epidemiological evidence linking Opisthorchiidae infection and hepatobiliary pathology, our understanding of the mechanistic and descriptive biochemistry of the association is still rather poor. Only the humoral response to *O*. *viverrini* is reasonably well documented; for instance, a study on the infected hamsters showed that a titer of the parasite-specific IgG in the acute stage is directly correlated to the intensity of infection, as determined by both worm burden and eggs per gram (EPG) counts. A more discriminative approach where the pathogen specific antibodies were divided according to their specificity to egg, excretory–secretory and somatic antigens, shows an interesting kinetic of humoral response: while in the acute phase of infection the antibody response was higher in the animals infected with higher dose of metacercariae, in chronic phase higher responses, particularly to somatic and egg antigens, were found in the lightly infected hamsters [9]. A recent report of Khoontawad *et al* [10] represents the most detailed proteomics work on *O*. *viverrini* infection published so far. The authors explore the differential protein expression in the host tissue with a chronic inflammation versus the tissue samples from the subjects with *O*. *viverrini* induced cancer. With regard to the metabolomics, a post-genomic discipline aiming at studying the metabolites—the end points and the intermediate products of the metabolism, the data is simply not available. The metabolome of body fluids is the closest approximation of the physiological phenotype of an organism and, as such, it represents an important, but still undervalued, source of clinical/physiological information [11]. The dynamic character of the metabolome, its ability to change in response to the external stimuli makes it an optimal “readout” for exploratory studies describing the systemic responses of an organism. Whereas in schistosomiasis a number of exploratory metabolomics studies have been performed in animal models and patient material[12–16], there are so far no such studies in Opischthorchiasis.

Here we present the first exploratory metabolomics study of an experimental model of *O*. *felineus* infection. Using an established hamster infection model we conducted a nuclear magnetic resonance (NMR) based metabolomics study involving a cohort of 30 animals with two degrees of infection (severe and mild) and a control group. Urine samples were collected every two weeks for a period over several months. Using a combination of unsupervised and supervised multivariate statistical analysis we were able to discriminate the time-resolved urinary metabolic patterns of the infection.

## Material and methods

### Ethics statement

All hamsters used in the study were handled according the recommendations of the national guidelines for animal caring: 12.08.1977 N 755 "On measures to further improve the organizational forms of work using experimental animals". The study was approved by the local ethical committee of Siberian State Medical University with a license number 3296 issued on 29.04.2013.

### Experimental opistorchiasis model

Hamsters *Mesocricetus auratus* were purchased from the animal facility of the Institute of Bioorganic Chemistry Academicians M.M. Shemyakin and U. A. Ovchinnikov. Metacercariae of *O*. *felineus* were obtained from naturally infected fishes sold in the local supermarkets as a consumption product. The muscular tissue and the subcutaneous tissue were digested by pepsin-HCl and viable metacercariae were collected and identified by microscopy. Hamsters were divided in 3 groups: high intensity of infection (50 metacercariae /hamster), low intensity of infection (15 metacercariae/hamster) and uninfected (vehicle—PBS) groups. Each group consisted of 10 animals- five males and five females. Hamsters were housed in separate cages, maintained on a 12:12 light-dark cycle (0600–1800:1800–0600 hs), and provided food and water *ad libitum* for the duration of the experiment. Hamsters were approximately five weeks old at the time of infection. Animals were sacrificed after 46 weeks of infection Livers were collected at the time of the sacrifice to count the adult worms. Urine and feces were collected every two weeks, specifically at the weeks 0, 2, 4, 6, 8, 10, 12, 14, 16, 18, 20, 22, 24, 26, 28, 30 and 32 after infection. For urine and feces collection hamster were placed individually into sterile empty glass crater and observed until urine and feces pellets were generated. Urine and fecal samples were collected into individually labelled Eppendorf tubes, and transferred to a freezer (-80°C) for long term storage.

### Eggs counts

The mean number of eggs per gram of feces was calculated following the modified Kato method (Katz et al. 1972). The fecal sample from each animal in the examined time point were mixed by Mini-Beadbeater-16 (Bio-Spec) for 5 minutes. A sample 25 mg of the feces was weighed with an electronic scale (Sartorius type 1702, sensitivity 0.1 mg) immediately after homogenization. Two slides were prepared for each 25 mg of feces. The extrapolation of the egg counts per 25 mg sample to the eggs per gram values was done by a simple multiplication (x 40).

### Urine sample preparation

All chemicals used for the buffer solution were purchased from Sigma-Aldrich except for the ^2^H_2_O which was purchased from Cortecnet and the 3-(trimethylsilyl)propionic-2,2,3,3-*d*_*4*_ acid sodium salt (TSP) from Cambridge Isotope Laboratories Inc. 96-well plates and NMR tubes were purchased from Bruker Biospin Ltd. (Germany).

Aliquots of 0.5 ml urine per sample were thawed overnight at 4°C. Cellular components and other insoluble material were then spun down by centrifugation for 10 min at 3184 g and 4°C and the supernatants were transferred into 96-well plates. 270 μL of urine from each sample were mixed with 30 μL buffer solution in ^2^H_2_O (pH = 7.4) containing 1.5 M K_2_HPO_4_, 2 mM NaN_3_ and 4 mM of TSP-2,2,3,3-*d*_*4*_ as an internal standard and chemical shift reference (0.4 mM final concentration in each sample). Finally, 165 μL of each urine-buffer mixture were transferred to 3 mm SampleJet NMR tubes and placed in refrigerated racks (6°C) of a SampleJet system until the NMR measurements. Both mixing of urine with buffer and transfer of the mixture to NMR tubes were performed by two 215 Gilson liquid handler robots and controlled by the SampleTrack software (Bruker Biospin Ltd.).

### NMR data acquisition

NMR data were recorded using a Bruker 14.1T AVANCE II spectrometer for ^1^H 600 MHz, equipped with a triple resonance inverse cryoprobe (TCI). Each sample was allowed to sit in the probe for 5 min to adopt a stable temperature at 27°C before starting the calibration routines and data acquisition. The probe was then automatically tuned and matched, followed by shimming and proton pulse calibration. One-dimensional (1D) ^1^H NMR spectra were recorded using the first increment of a NOESY pulse sequence[17] (noesygppr1d in Topspin 3.0 library). Water signal suppression was achieved with pre-saturation, a continuous wave irradiation of 50 Hz soft pulse during the relaxation delay of 4 s and the mixing time of 10 ms. The spectral width was set to 20 ppm (12335 Hz) and 16 scans of 65536 points were collected. The recorded free induction decays (FIDs) were Fourier transformed and a line broadening of 1.0 Hz was applied. The spectra were automatically phased and baseline corrected and referenced to the internal standard chemical shift (TSP; δ 0.0 ppm). An evaluation of spectra quality was performed after processing. Peaks line-width was evaluated with the TSP singlet and Alanine’s methyl protons doublet. In addition, the efficiency of water suppression and the quality of the baseline were also checked. Spectra which failed to fulfil the quality criteria were discarded from further analysis.

Two dimensional *J*-resolved spectra (2D *J*res) were also collected for each sample using the same water suppression scheme as described above during the relaxation delay of 2 s. The spectral width was set to 16.66 ppm (12288 Hz) for the direct dimension and 78 Hz for the indirect one and 2 scans were acquired over 40 increments. The FIDs were automatically processed with Fourier transformation and spectra were referenced to the TSP signal at 0.0 ppm in the F2 dimension and at 0.0 Hz in the F1 dimension.

For assignment purpose, 2D NMR spectra were also acquired for a sample is made as mix all urine samples. The set of 2D experiments included ^1^H-^1^H correlation spectroscopy (COSY), ^1^H-^1^H total correlation spectroscopy (TOCSY), ^1^H-^13^C heteronuclear single quantum correlation (HSQC) and ^1^H-^13^C heteronuclear multiple bond correlation spectroscopy (HMBC) using the standard parameters implemented in Topspin 3.0 library (Bruker Biospin Ltd.).

### Spectral data processing and data analysis

Pre-processing of NMR data (S2 File) to be suitable for statistical analysis was performed with in house routines written in Matlab 2014a (The Mathworks, Inc., USA) and Python 2.7 (Python Software Foundation, www.python.org). All 1D NMR ^1^H spectra were re-evaluated for incorrect baselines and corrected using a polynomial fit of degree 5. The spectral region from 0.5 to 9.5 ppm was binned using an in-house algorithm for adaptive intelligent binning[18]. Initial bin width was set to 0.02 ppm and final variable bins sizes were calculated based on the peaks edges in the spectra by using a lowest standard deviation criterion. The spectral region including the residual water and the urea peaks (δ 4.5–6.2 ppm) was excluded from the data.

The final data consisted of 392 bins of variable size × 490 observations (samples), which were normalized by the Probabilistic Quotients Normalization method (PQN)[19] to correct for dilution differences from sample to sample. Finally, the normalized data was scaled to unit variance for the statistical analysis.

The data analysis was performed with R statistical environment ((http://www.r-project.org/, R versions 3.3.2). For exploratory analysis “*Rcpm*”, “*pcaMethods*” and “*caret*” packages were used. ASCA modeling was performed using “lmdme” package [20]. The visualizations were made using “ggplot2”, “*cowplot*” and “*gridExtra*” packages.

### Identification of the metabolites

Identification of metabolites was performed by exhausting search of the total 1D and 2D *J*res data using the proprietary Bbiorefcode (Bruker Biospin Ltd.) and ChenomX NMR suite 8.1 (Chenonx Inc.) databases. The IDs of the annotated resonances were further verified by the collected 2D NMR data.

## Results

### Adult worm and eggs counts

**Fig 1:**
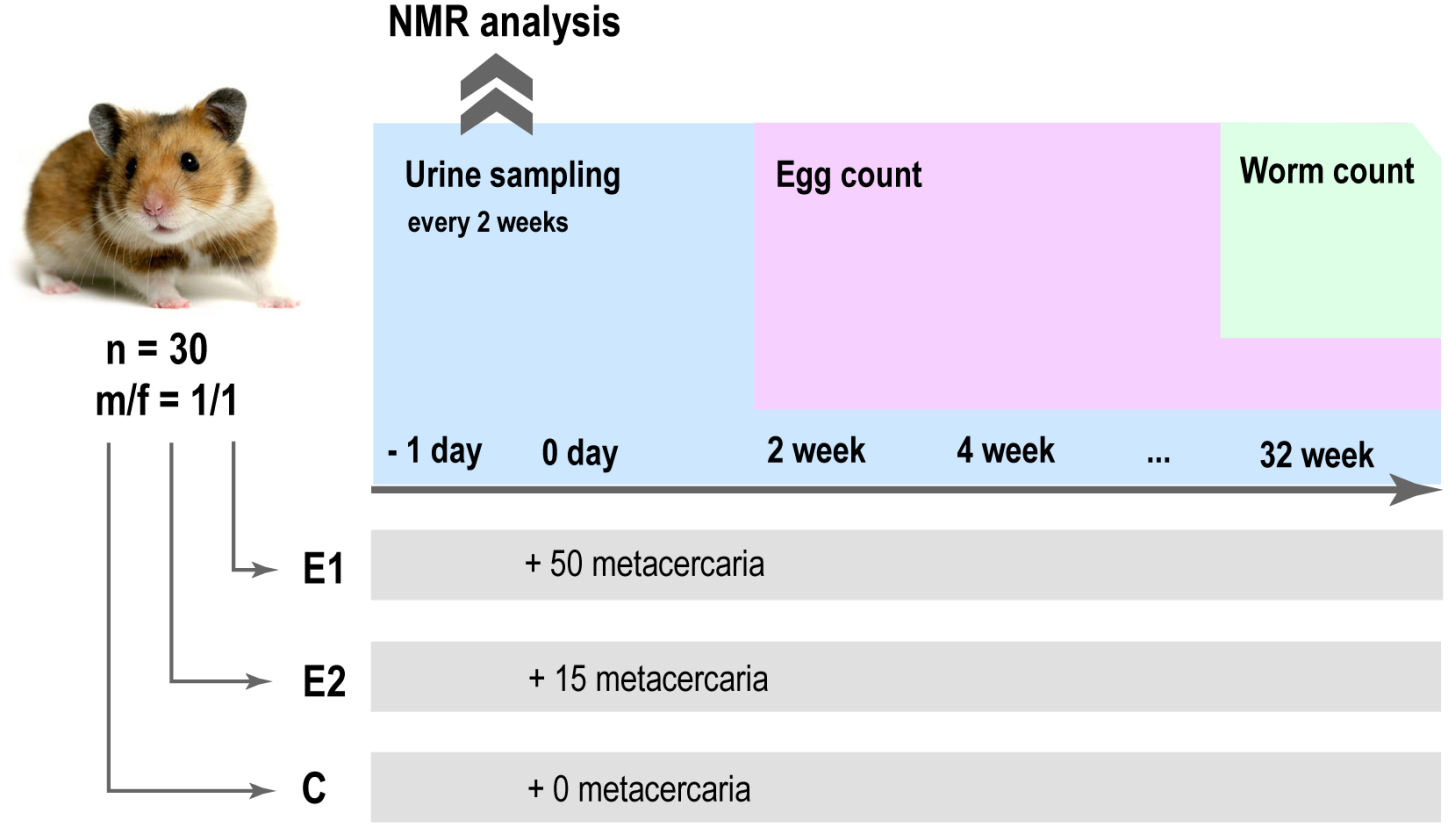
A graphical outline of the experimental design.

The study design involves 30 animals divided in three groups: a control uninfected group and the two experimental groups infected with fifteen and fifty metacercaria Fig 1. Each experimental group consisted of an equal number of male and female animals. The dataset described in the current manuscript includes the samples from the baseline up to the thirty-two weeks collected every two weeks. The median of adult worm count at the end of the study was 35 for severe and 6 for mild infection intensity group respectively (p-value = 0.003) (S1A Fig). Eggs of *O*. *felineus* were detected in all fecal samples starting from 4 weeks post infection. S1B Fig shows the time course of the egg production over the entire period of the experiment. It shows a coherent increase in the egg production for both experimental groups over a period from 4 till 10 weeks. We consider changes in the egg production related to the different stage of the infection, namely an acute (till 10 weeks) and chronic one (from 10 weeks on). From week 12 till week 30 of the experiment, egg output is stable and the group with high infection has constantly higher egg output. The output is significantly higher at the weeks 12 (p-value = 0.002), 14 (p-value = 0.004), 16 (p-value = 0.030), 20 (p-value = 0.001), 26 (p-value = 0.001) and 28 (p-value = 0.009).

### An exploratory analysis of urinary metabolic profiles

**Fig 2:**
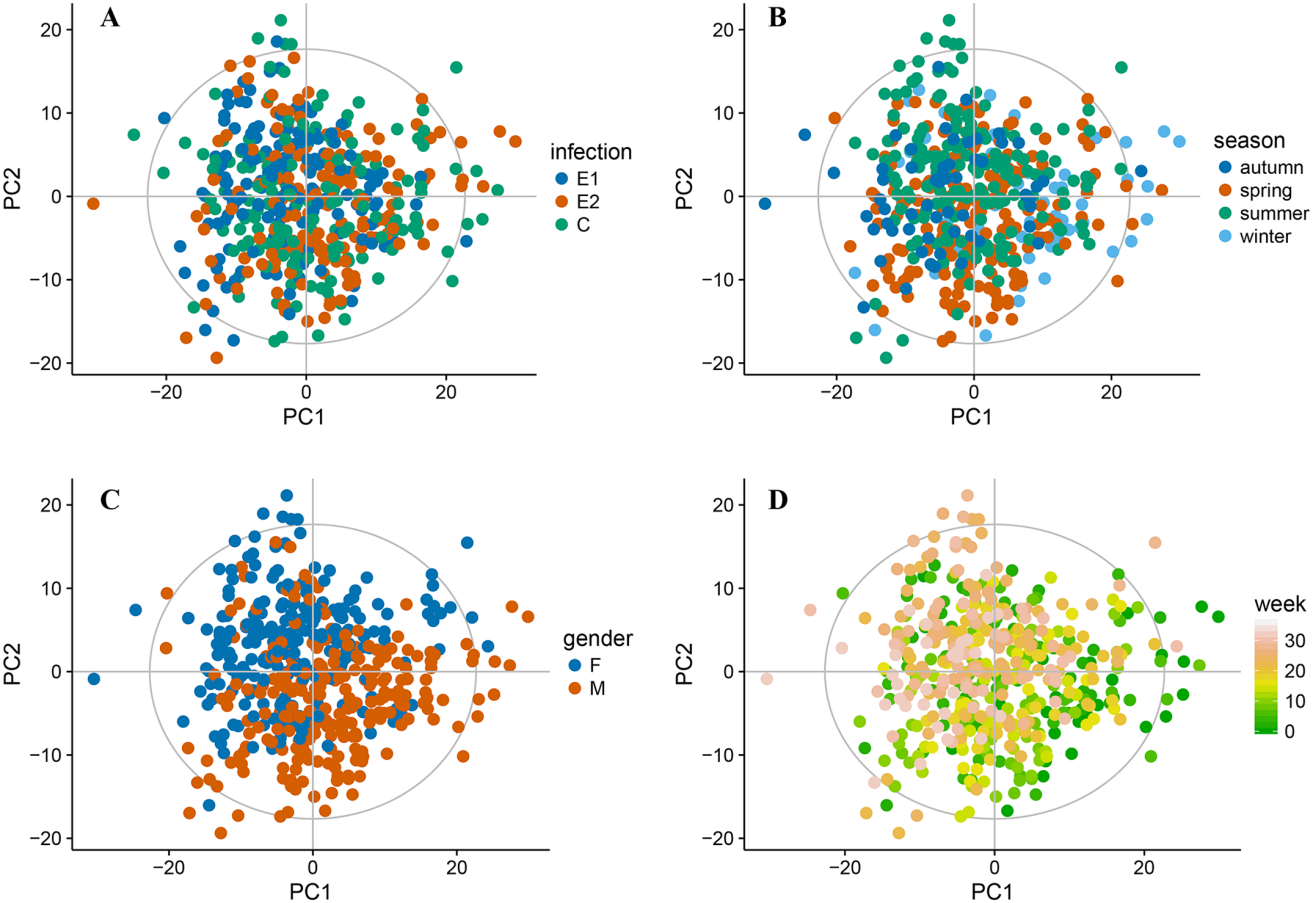
The score plots of the first two principal components of a PCA model built on the entire dataset; Every block of the figure represents the same model colored according to different factors; A—infection group, B—season, C—gender, D—time in weeks.

The first step of an exploratory study is to identify the main sources of the variance in the data and influence of the confounding factors. The principal component analysis (PCA) is a commonly accepted approach. Fig 2 shows the score plots of the first two principle components of a PCA model built on the entire data set. The model required 5 components to explain 50% of the variance with 34% explained by the first two components. Every block of the figure represents the same model colored according to different factors. Surprisingly, the intensity of the infection (Fig 2A) is not contributing to the main sources of the variance in the data. Gender influences the profile profoundly, the influence is clearly represented in the first two principal components (Fig 2C). Another recognizable source of the variance is the time of sample collection (Fig 2B and 2D). The S2 Fig shows the PCA models built separately for male (A, B) and female (C, D) animals. The models have similar characteristics to the “global” one, both require 5 components to explain 50% of the variance; the first two components explain 37% and 32% of the variance in the male and female models, respectively. Again the intensity of infection is not explaining the main variance within the data (S2A and S2C Fig), but the time trend is clearly visible (S2B and S2D Fig). Moreover, the directions of the time trends in the models built on gender specific subsets appear to be different.

### Analysis of the gender specific metabolic changes in the acute phase of the infection

**Fig 3:**
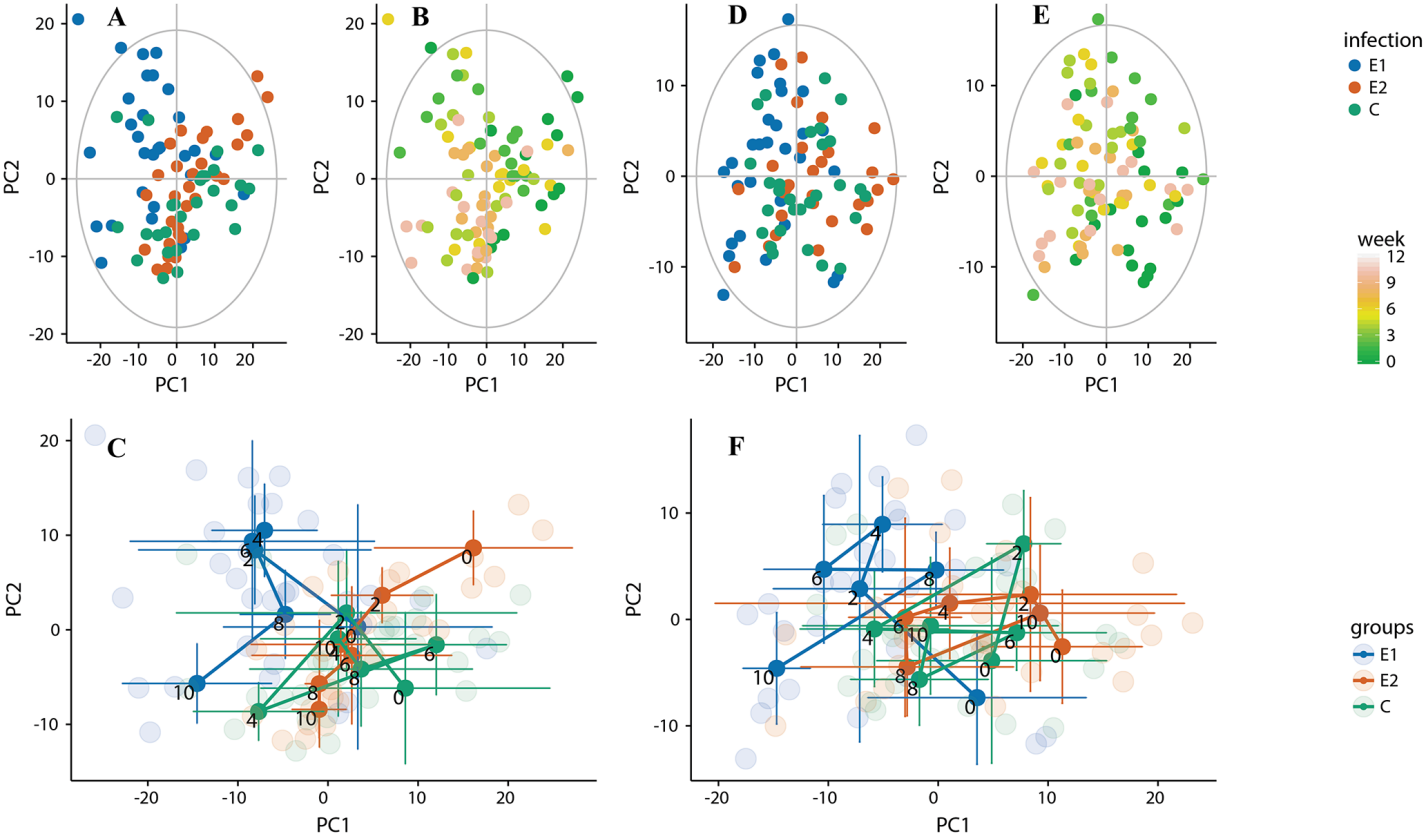
PCA models build for each gender group separately on a subset of the samples collected between 0 and 10 weeks; A, B—males, D, E—females. Geometric time trajectories: C–males, F—females.

The data shown in Fig 2 indicates clearly that a straightforward analysis of the infection dependent changes will be hampered by the influence of the gender and time trend. Thus, to reduce the complexity of the analysis we decided to concentrate on the period from 0 to 10 weeks of the experiment: a time frame when egg output was steadily increasing (S1B Fig). The analysis was performed separately on the male and female subsets. Fig 3 shows the PCA models built on the data from week 0 up to the week 10 of the experiment. The “male model” (Fig 3A, 3B and 3C) required four components to explain 50% of the variance with 42% explained by the first two. The model build of the data including only females (Fig 3D, 3E and 3F) required 6 components to explain 50% of the variance with the first two explain 34%. Both models appear quite similar, however applying the geometric trajectory analysis [21] (Fig 3C and 3F) some underlying differences can be revealed. The geometric time trajectories for the infected animals (groups E1 and E2) are clearly pronounced in the male model (Fig 3C). The same time in the female subset (Fig 3F) only a trajectory for highly infected animals (E1) has a distinct form, a trajectory for the lower infection group appears as random as the control one.

### ANOVA simultaneous component analysis (ASCA) modeling

**Fig 4:**
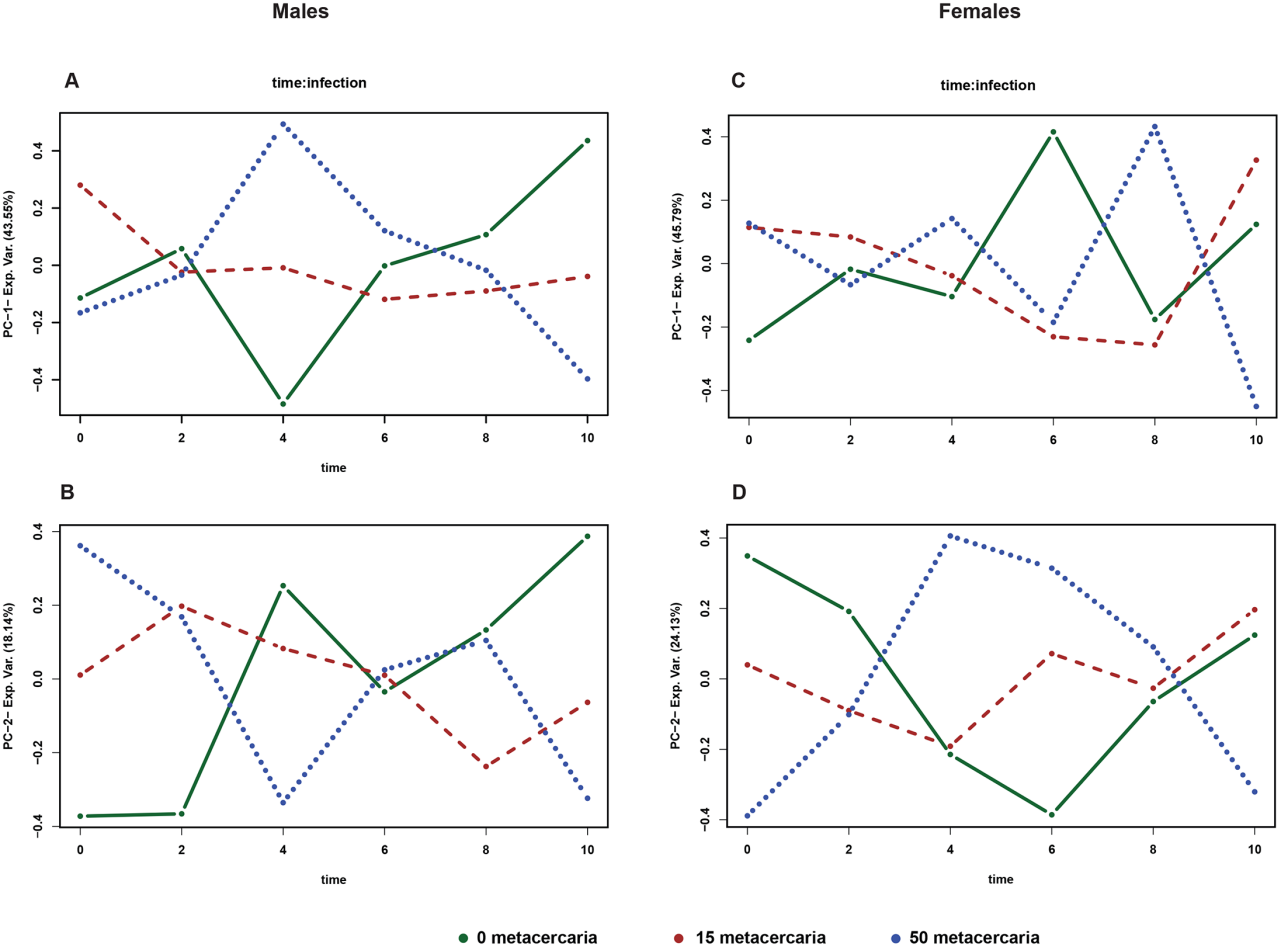
ANOVA simultaneous component analysis loadingplot on metabolites satisfying the F test with p value < 0.005 on the interaction coefficients: Time × infection. A, B—males, C,D—females.

To dissect the metabolic features related to the infection one needs a data analysis approach which enables simultaneous analysis of the experimental design and time. The choice is rather limited. One of the “stress tested” methods is ANOVA simultaneous component analysis or ASCA [22, 23]. In a nutshell, ASCA is a multivariate extension of the ANOVA and the strongest asset of the method is a possibility of modelling of the experimental designs with several factors. With regards to our study those factors are the time, intensity of the infection and gender. To avoid a complex interpretation of the multiple cross-factor interactions we applied ASCA separately to each gender subset. Fig 4 shows the results of the analysis for the first two components. The trends are looking similar, but not identical. In the male subset the infection specific time trends are present in the main component (43.5% variance). The time trends for non-infected and infected with 50 metacercaria animals have almost opposite behavior in the first component showing somewhat random pattern in the second one. In the female subset, the infection related trends are not presented in the first component, but well presented in the second one. The last one, however, covers only 24% of the variance, almost the half of what was seen in males.

### Selection and annotation of the variables

Fig 4 shows the time/infection specific trends and the systemic differences between the males and females in the reaction on the infection challenge. Yet, it gives no information on the nature of the biochemical entities involved in the observed phenomena. Thus, using a leverage as a measure of the variable importance we extracted a subset of the variables influencing the models shown at Fig 4. Table 1 summarizes the selected variables. Most of the variables can be annotated, but few complex regions (1.63–1.69, 1.71–1.74 and 2.46 ppm) simply cannot be assigned to a single metabolite. S1 File shows the 24 week time trends of the every individual feature included in the table. The traces are plotted as the median values per experimental group, the original values are included in the plots in a form of transparency graphics. A visual inspection of the graphs shows that only few compounds demonstrate a consistent trend over the entire period. For most of them, the changes are evident only in the early weeks; some compounds (e.g. 2−aminoadipic acid and nicotinuric acid) shows a distinct trend for the group of the animals infected with 50 metacercaria.

**Table 1:** The annotation of variables selected on basis of the ASCA modeling.

	Bin	Compound ID*	Gender group**
1	**0.7198**	**Bile acids**	**f, m**
2	**0.8924, 0.929**	**Pantothenic acid**	**m**
3	**1.0258**	**L-Isoleucine**	**m**
4	**1.6366, 1.6562, 1.6798, 1.6942**	**5-aminopentanoic acid & 2-aminoadipic acid**	**f**
7	**1.7138, 1.7386, 1.7472**	**Leucine, Lysine**	**f**
10	**1.833**	**Crotonic acid**	**m**
11	**2.4634**	**3-(3-hydroxyphenyl) propionic acid, hydroxyferulic acid, glutamine & pyridoxine**	**f**
12	**3.4394**	**taurine, glucose**	**m**
13	**3.6704, 7.4332**	**phenylacetylglycine**	**m**
14	**3.7128**	**threitol**	**m**
15	**3.7872**	**guanidinoacetate**	**m**
16	**6.4**	**urocanic acid**	**m**
17	**6.78**	**homovanillic acid**	**f**
18	**6.8798, 6.8948**	**tyrosine**	**f**
19	**6.9596**	**hydroferulic acid**	**f**
20	**7.029**	**5-hydroxyindoleacetic acid**	**f**
21	**7.34, 7.3728, 7.4**	**phenylacetate**	**m**
22	**8.6798**	**nicotinic acid**	**f**
23	**8.7**	**nicotinuric acid**	**f, m**
24	**8.7282**	**nicotinamide-N-oxide**	**f, m**

* compound(s) ID protons of which resonate within the selected bins

** column shows whether a bins were selected from model built on the male or female subgroup (m—males, f—females).

## Discussion

Here we present for the first time an exploratory analysis of the metabolic response to *O*. *felineus* infection in an animal model. Following an established routine of a descriptive study we started the analysis by exploring the main sources of the variance in the data. Using unsupervised projections methods namely PCA we have shown that neither infection status nor the time are representing the major sources of the variance in the data. In fact, the most noticeable trend in the data was related to the gender differences. At first glance, this observation may appear contradictory to the earlier publications on the animal models infected with other trematodes *S*. *japonicum*[13] and *S*. *mansoni* [12] as well as to our own report on a human study[15] where clear differences between infected and non-infected subjects were reported. Yet, to reveal the infection related differences authors of the above mentioned studies had to use the supervised modeling techniques and an unbiased assessment of the main sources of the variance in the data as a rule showed the influence of the physiological confounding factors such as for example age [15, 24]. A gender related bias in human studies was concealed by age factor due to the broad age range of the participants. In the animal studies the single gender models were used: the female animals for mice [12, 14, 25] and the males for hamsters [13, 26]. Since the animals included in our study were age matched, gender is now the strongest physiological cofounder [27]; consequently the gender bias observed in the data should be expected. Of course one should keep in mind the all the above metioned examples are taken from the publications on the metabolic effects of *Schistosomiasis*, no information on *Opisthorchiasis* has been published yet.

A gender related trend explains approximately 30% of variance in the data; therefore further analysis was done of each sub-group separately. Both unsupervised multivariate modelling and ASCA-based analysis showed that a time related response to the infection unfolds differently in the males and females. Moreover, the Fig 4 which provides an overview of the main patterns associated with time-infection interaction clearly shows that in the male model the distinct trend is present in the first principle component covering twice as much variance as the female model where the similar trend is visible only in the second component. Naturally, a discussion of the biological relevance of the observed effects is only possible if we know the identity of the metabolites influencing the models. A subset of the most influential entities is presented in the Table 1. In overall it appears as a more or less standard set of the metabolites which are regularly reported in the NMR-based metabolomics studies of urine. It also has a strong overlap with the metabolites reported in the publications on *Schistosomiasis* mentioned above.

In the context of an acute *Opisthorchiasis* infection, a state of the energetic stress, one particular trend, namely the bile acids becomes interesting. S1 File shows that in the group of heavy infected animals urinary excretion of the bile acids is increasing staring from the beginning of the experiment reaching the peak at four weeks and going down after that point. A current physiological paradigm links an increased urinary excretion of the bile acids with the possible obstructions of the main duodenal path [28]. We can only speculate about the exact physiological mechanisms which leads to the increase of the bile acids secretion but the timing of the observed effect overlaps perfectly with a period when the parasite “settles down” in the host’s bile ducts. Of course our method catches only a gross effect and cannot provide the exact annotation of the bile acids. From the literature we know that the bulk of the urinary bile acids are excreted as the sulphonated species [29] but to our best knowledge there is no published report on urinary bile acids profiles in *Opisthorchiasis*. A similar transient pattern to the bile acids was also observed for the spectral areas where resonate lysine and a product of its catabolism 5-aminopentanoic acid are located. Despite an intriguing similarity, a “simple” interpretation where lysine would be considered as a breakdown product of the conjugated bile acids is difficult to accept; the lysine conjugates are rather unusual and were reported only as the minor products of lithocholic acid in the liver [30]. Another group of the metabolites which are related to the physiological control of the lipid metabolism consist of nicotinic acid and its metabolites nicotinuric acid and nicotinamide-N-oxide. The last two compounds show a transient decrease with a peak at four weeks. The effect is more pronounced in the male group. It is logical to assume that the host energy metabolism works in a stress mode during invasion of the parasite the bulk of nicotinic acid pool will be utilized for NAD+ production, in turn this can lead to a reduced production and urinary clearance of nicotinuric acid and nicotinamide-N-oxide. Another metabolites which nicely fits into a pattern of the stress mode of the energy metabolism is guanidinoacetate—a precursor of creatine [31]. Of course, an interpretation of the data from a point of view of the metabolic stress has a weakness. None of the described changes can be considered as specific for *Opisthorchiasis*; all the changes reflect a general reaction of an organism to acute infection. Yet, an exploratory study, especially the one that enters a relatively uncharted territory should provide the leads and hypothesis for further investigation. To this end, we have fulfilled a purpose of the study. A logical follow up of our study appears to be an in depth analysis of the urinary and fecal profiles of the bile acids using more sensitive techniques namely mass spectrometry. Already the last decades of twentieth century the analytical methods enabled detection and quantitation of several of the bile acids [32], the state of the art technology offers a reliable analysis of more than hundred species [33]. Besides, the existing reports on alteration in the urinary bile acids profiles during the pathological conditions of liver [34] supports our hypothesis. Considering the other mentioned compounds and trends they fit a context of the energetic stress and metabolic redistribution of the bulk metabolites in the circulation, it remains to be seen however whether our findings can be translated to the human infection.

## Supporting information

S1 Fig An overview of infection intensity. A. Worm count at the end of the experiment; B. Time dynamics of egg output.
(PDF)

S2 Fig PCA models built separately for male (A, B) and female (C, D) animals. (PDF)

S1 File Time trajectories for the metabolites included in the Table 1. (PPTX)

S2 File The binned data table. (XLSX)
