# Supplementary figures and images for: Exploratory metabolomics study of the experimental opisthorchiasis in a laboratory animal model (golden hamster, Mesocricetus auratus)
Source: PLoS Negl Trop Dis. 2017 Oct 31;11(10):e0006044. doi: 10.1371/journal.pntd.0006044 (PMC5681294; doi:10.1371/journal.pntd.0006044)

**Worm count at the end of the experiment**

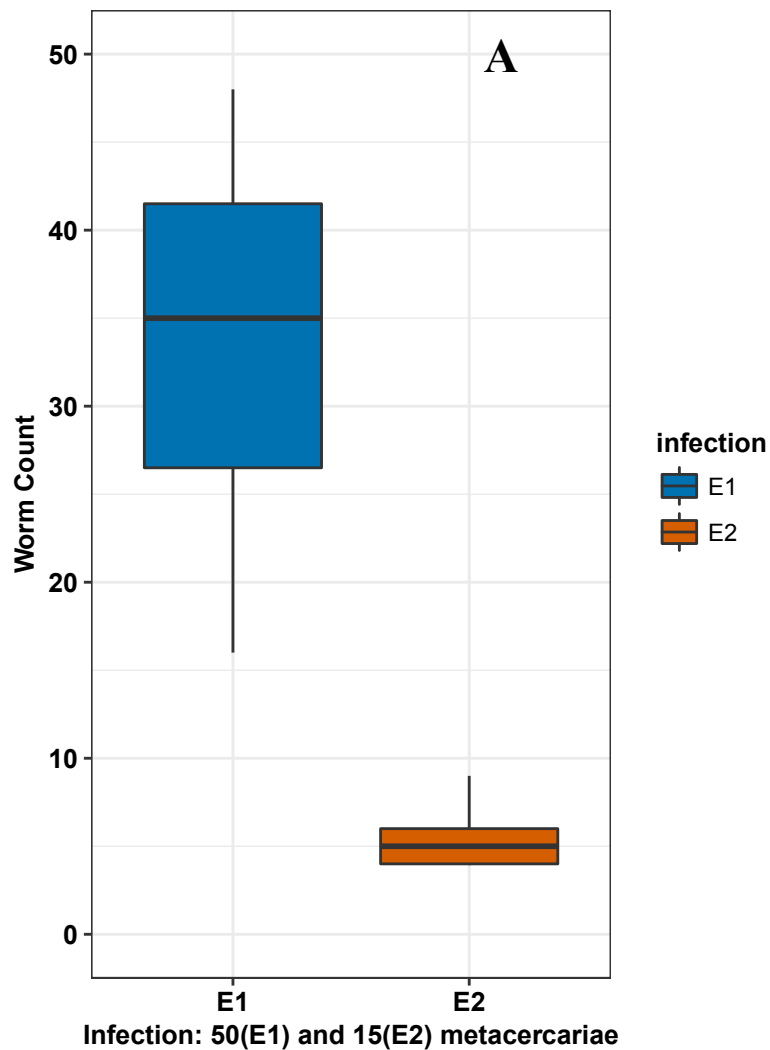

**Time Dynamics of Egg Output**

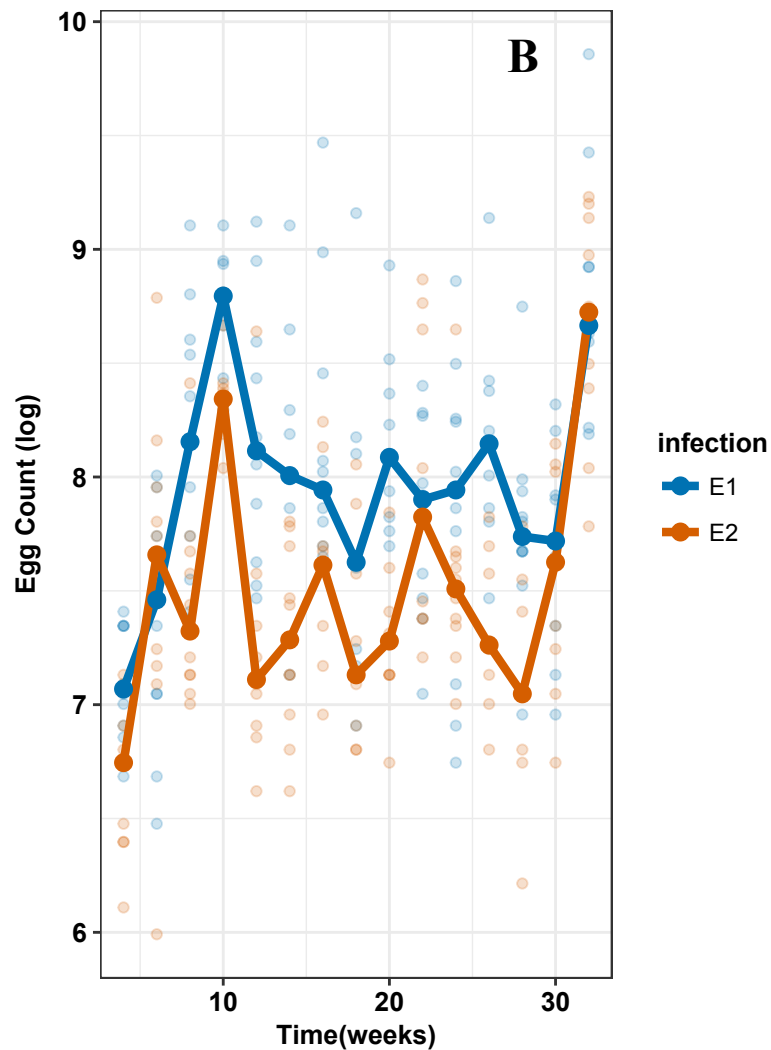

Supplement: S1 Fig — A. Worm count at the end of the experiment; B. Time dynamics of egg output. (PDF) [file pntd.0006044.s001.pdf]

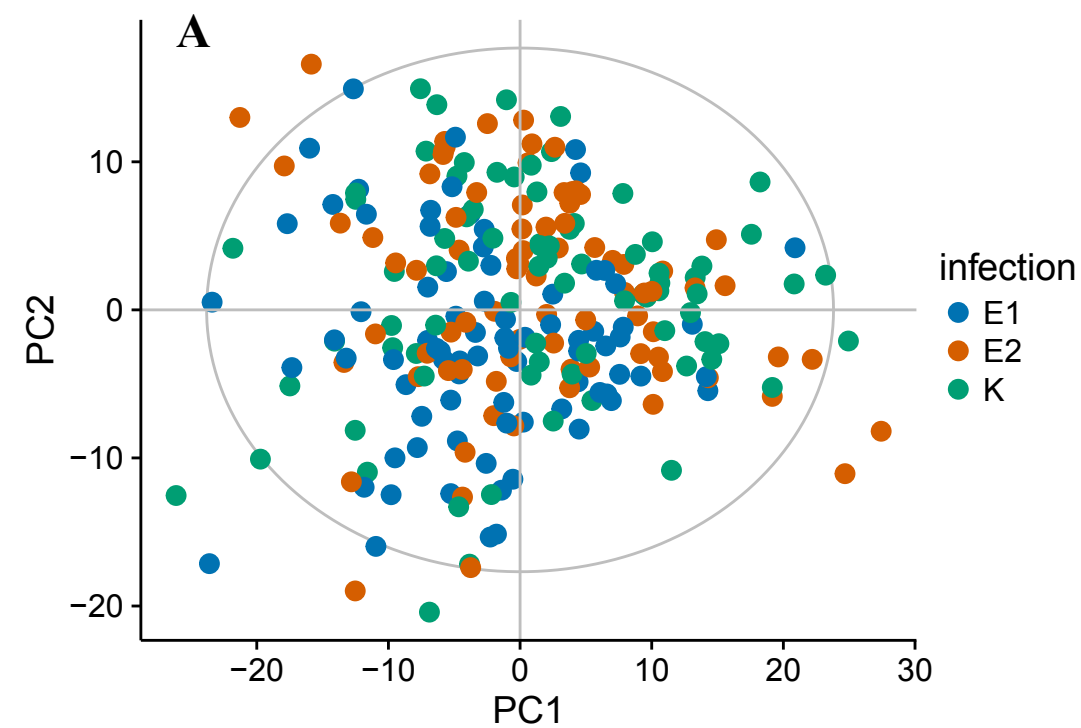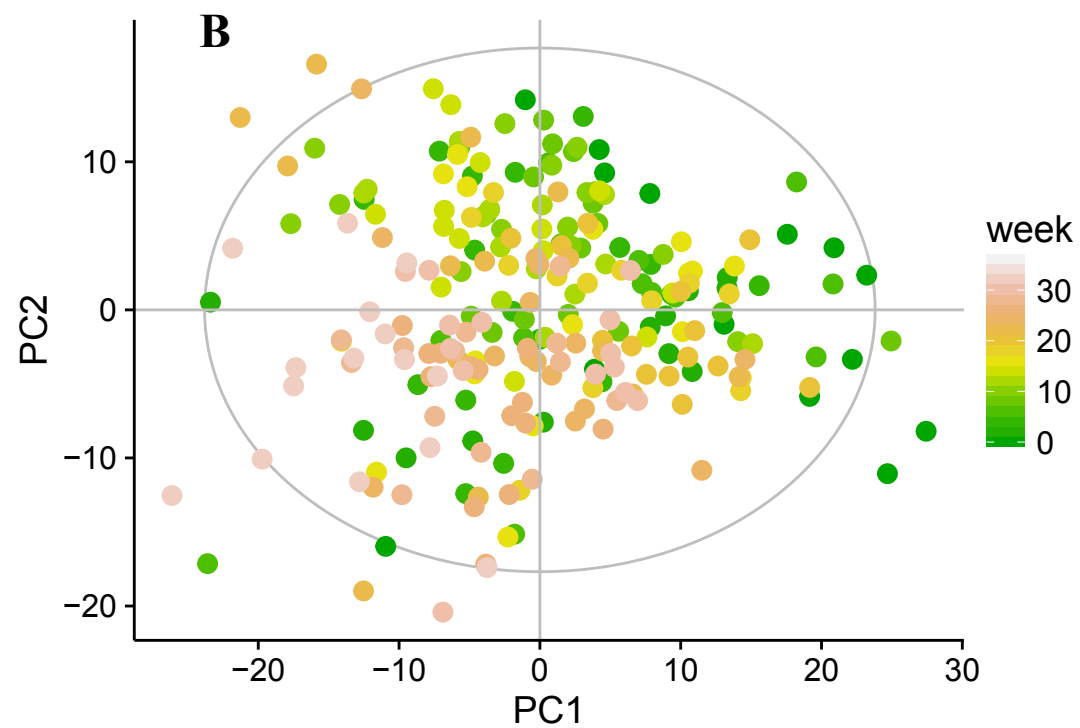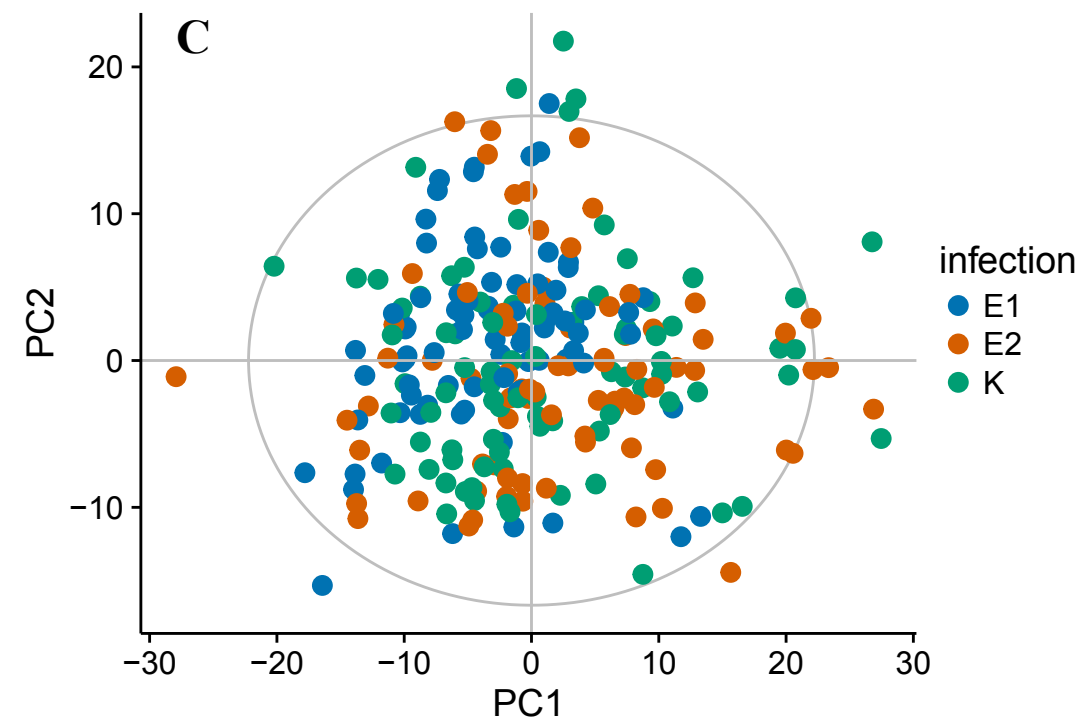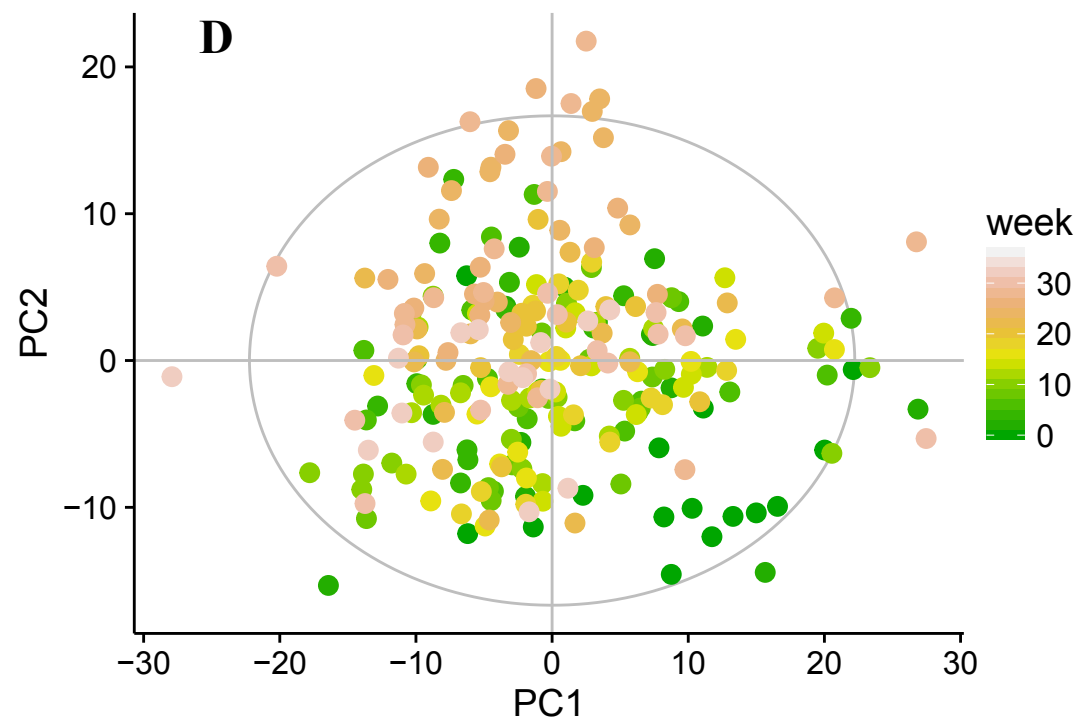

Supplement: S2 Fig — (PDF) [file pntd.0006044.s002.pdf]

## Slide 1
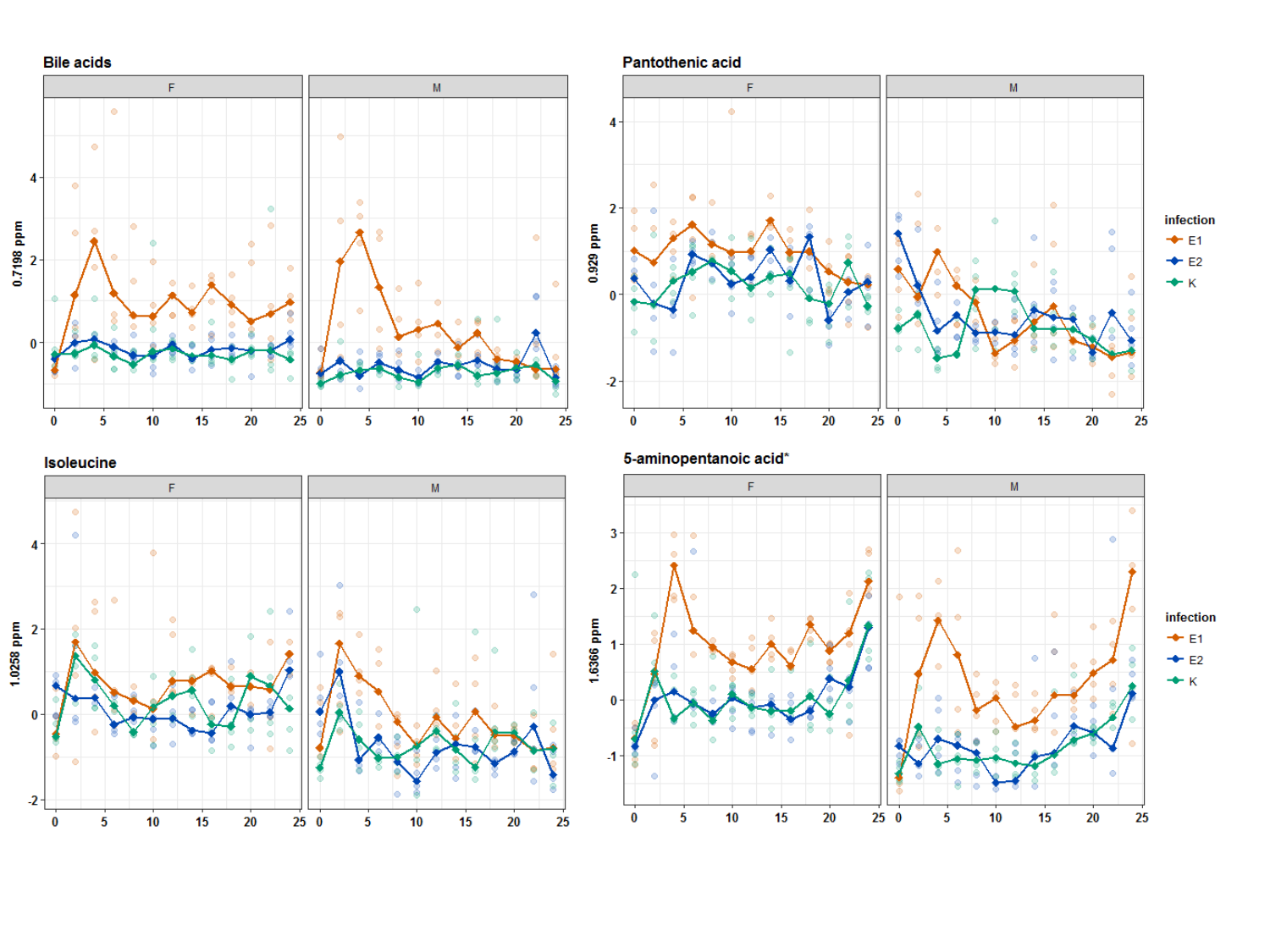

## Slide 2
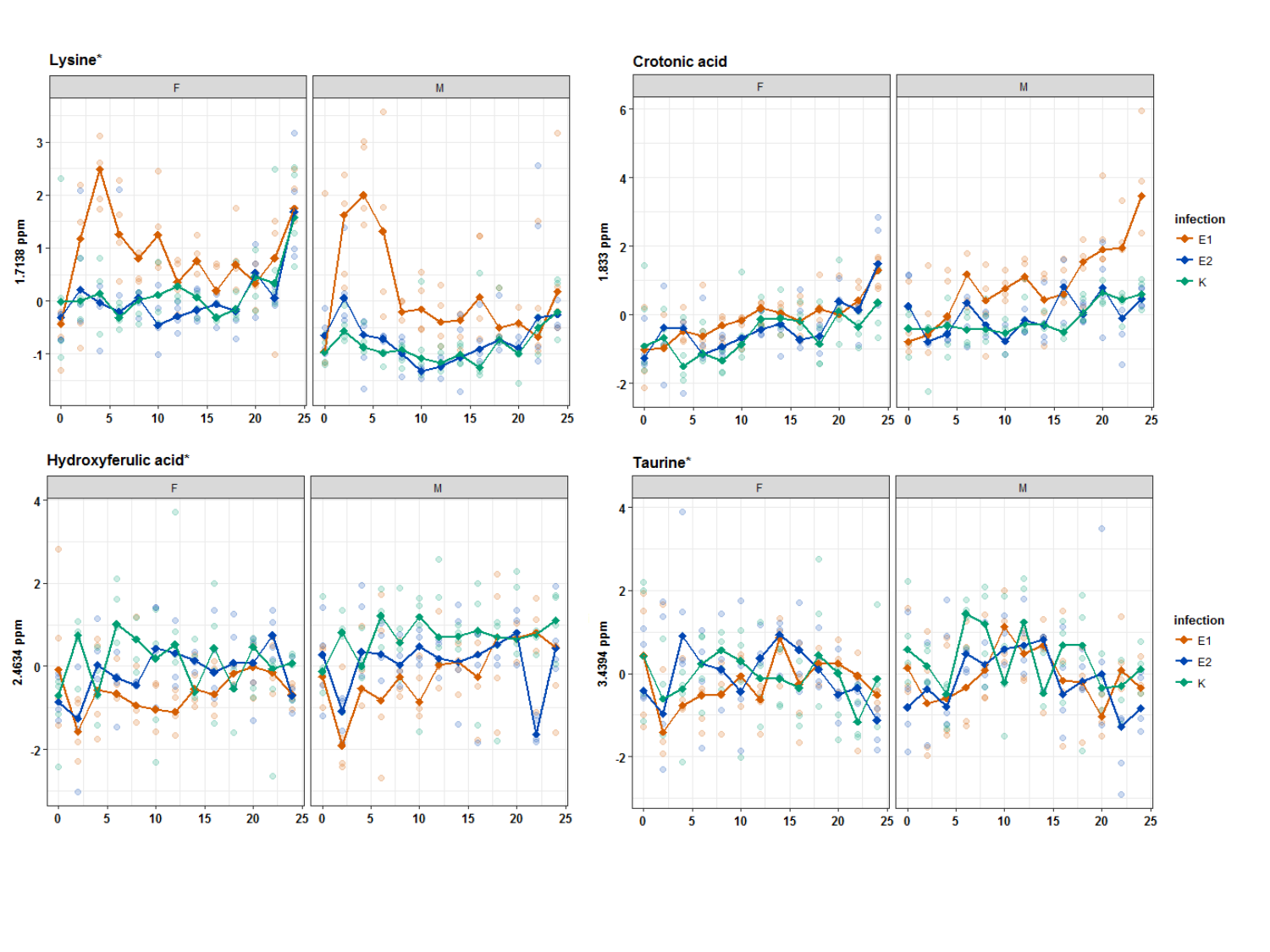

## Slide 3
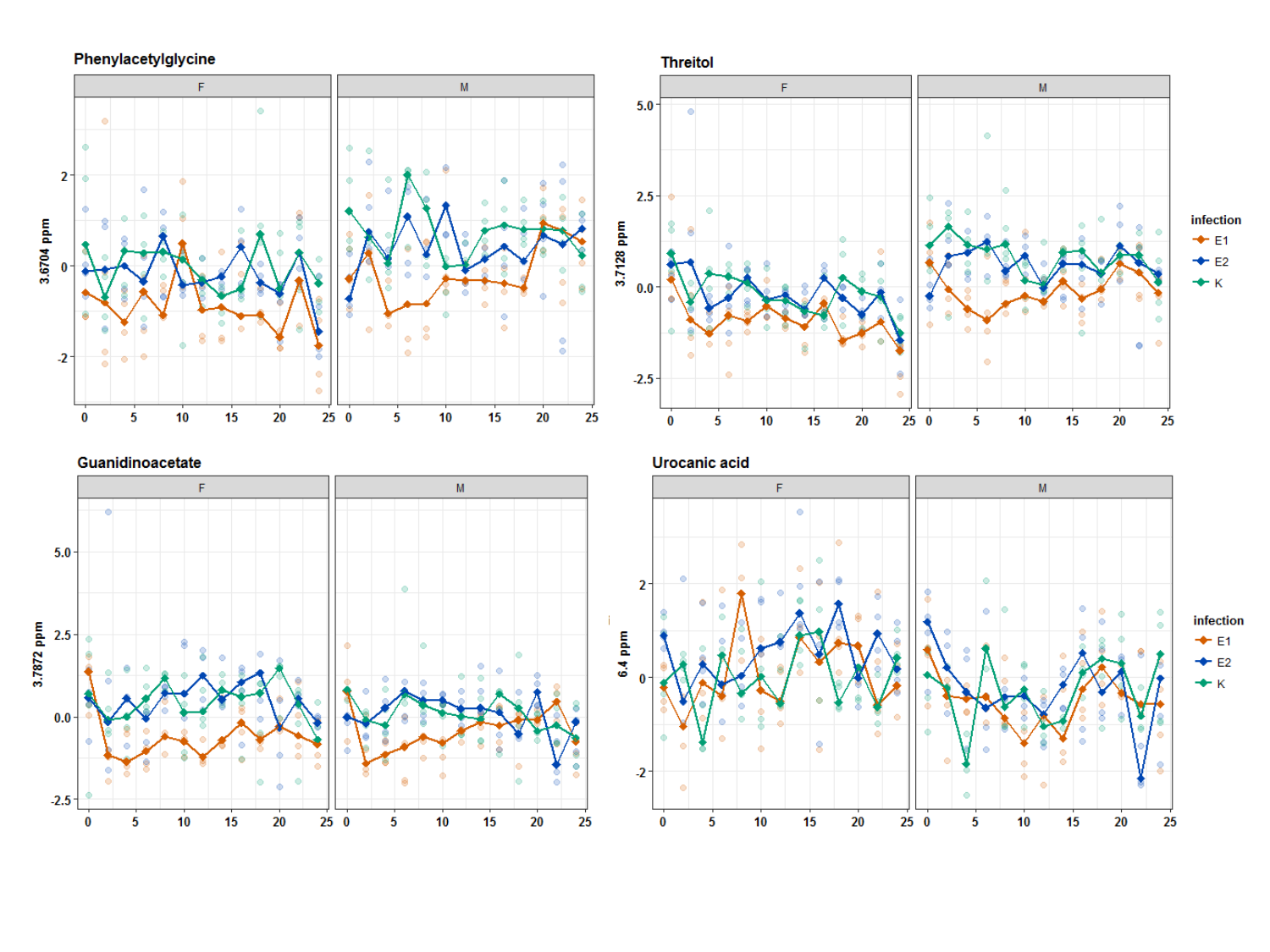

## Slide 4
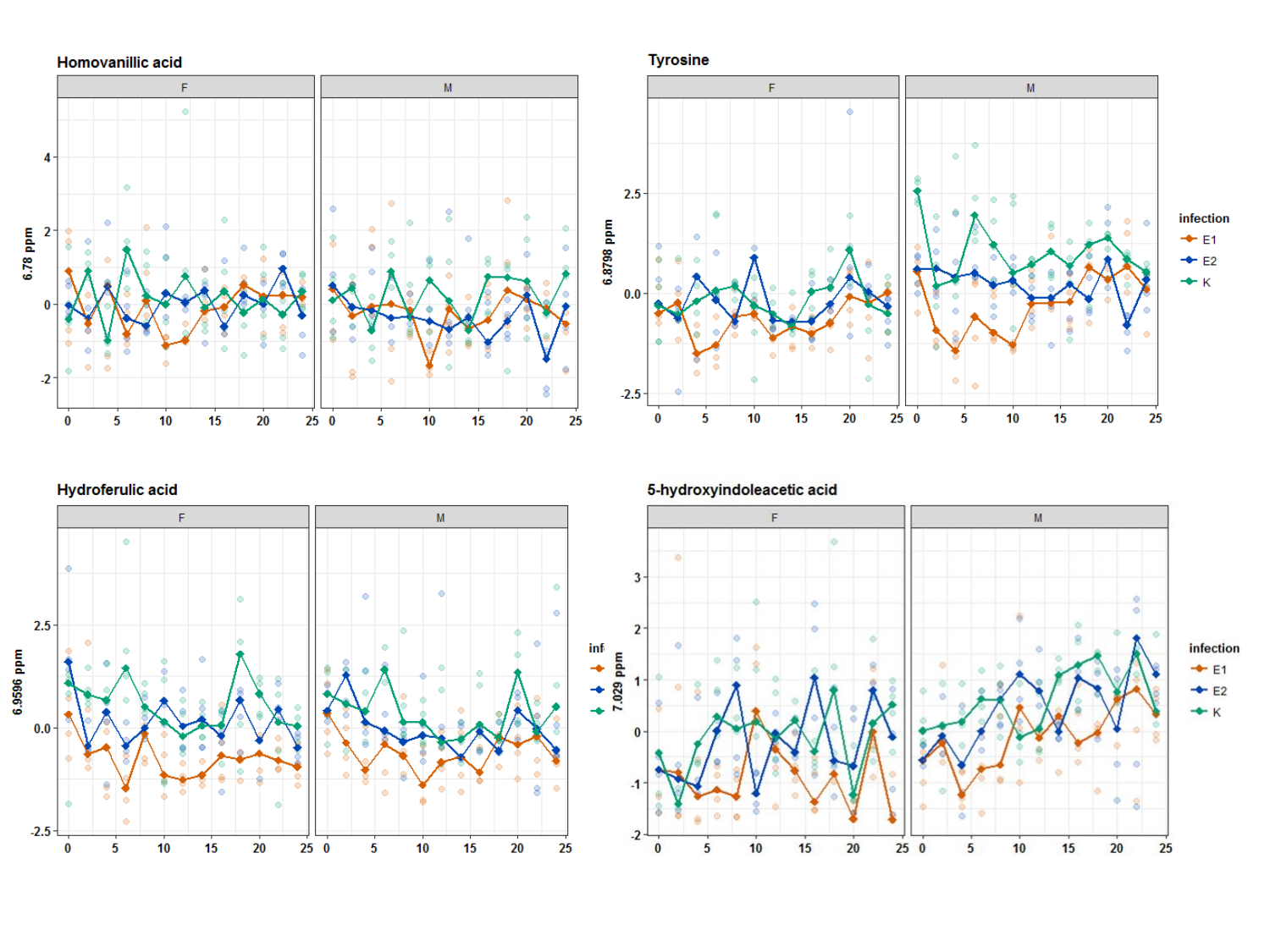

## Slide 5
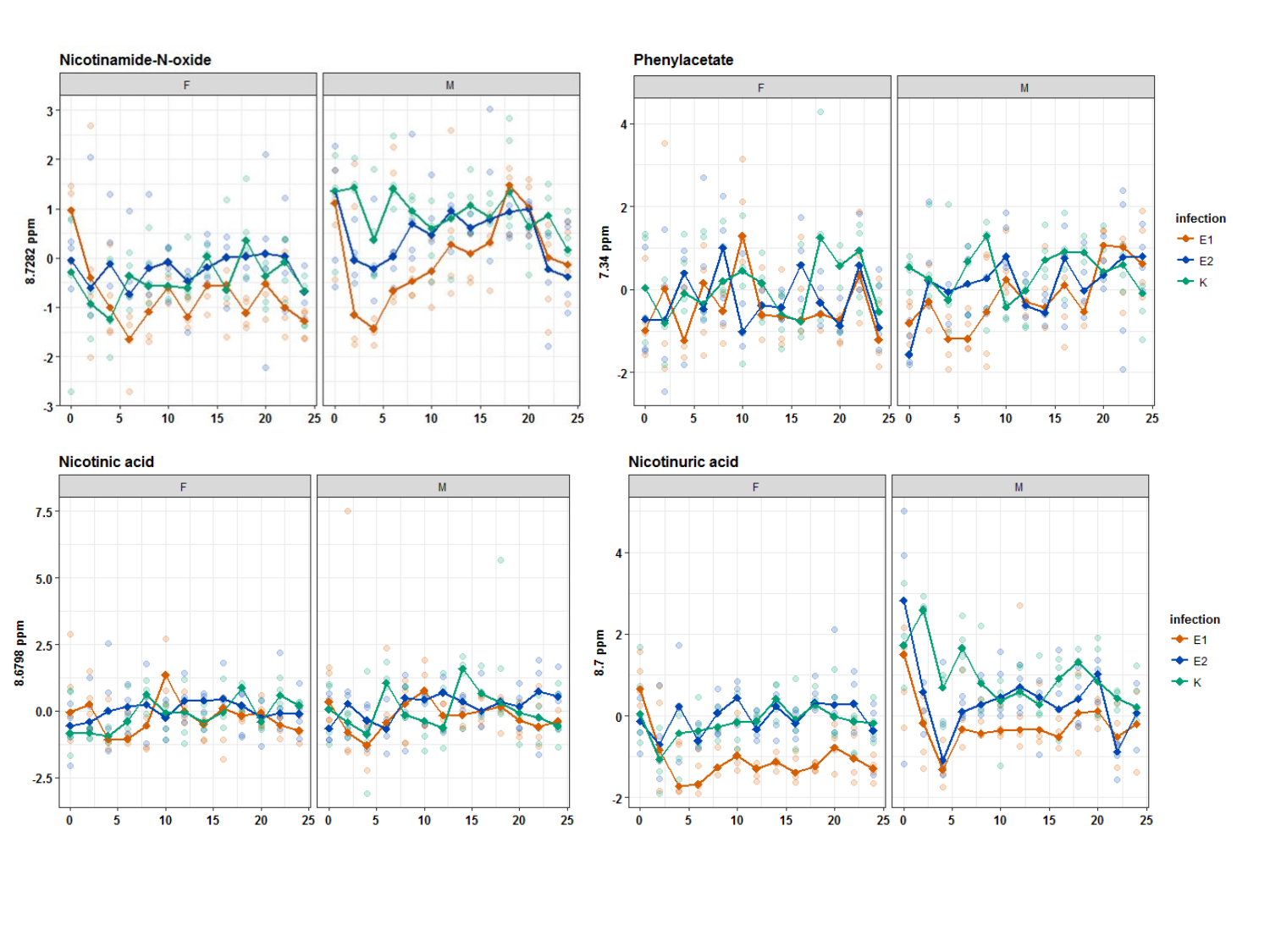

Supplement: S1 File — (PPTX) [file pntd.0006044.s003.pptx]
